# Transcriptome wide changes in long noncoding RNAs in diabetic ischemic heart disease

**DOI:** 10.1186/s12933-024-02441-6

**Published:** 2024-10-17

**Authors:** Amit Kumar Rai, Natarajaseenivasan Suriya Muthukumaran, Noemi Nisini, Tiffany Lee, Ioannis D. Kyriazis, Claudio de Lucia, Michela Piedepalumbo, Rajika Roy, Shizuka Uchida, Konstantinos Drosatos, Malik Bisserier, Rajesh Katare, David Goukassian, Raj Kishore, Venkata Naga Srikanth Garikipati

**Affiliations:** 1 Aging + Cardiovascular Discovery Center, Lewis Katz School of Medicine, Temple University, Philadelphia, 19140 USA; 2 Present Address: Laboratory of Biology, Faculty of Medicine, University of Thessaly, Larissa, Greece; 3 Present Address: ASL (Azienda Sanitaria Locale-Local Health Authority), Napoli 1 Centro, Naples, Italy; 4 ASL (Azienda Sanitaria Locale-Local Health Authority, Napoli 3 Sud, Naples, Italy; 5 Present Address: ASL (Azienda Sanitaria Locale-Local Health Authority), Salerno, D.S. 60, Nocera Inferiore, SA Italy; 6 Present Address: Department of Surgery, Duke University School of Medicine, Durham, NC USA; 7 Department of Clinical Medicine, Center for RNA Medicine, Aalborg University, Frederikskaj 10B, 2. (Building C), Copenhagen SV, 2450 Denmark; 8 Metabolic Biology Laboratory, Department of Pharmacology and Systems Physiology, Cardiovascular Center, University of Cincinnati College of Medicine, Cincinnati, OH USA; 9 Department of Cell Biology and Anatomy and Physiology, New York Medical College, Valhalla, NY USA; 10 Department of Physiology, HeartOtago, School of Biomedical Sciences, University of Otago, Dunedin, New Zealand; 11 Cardiovascular Research Institute, Icahn School of Medicine at Mount Sinai, New York, USA; 12 Department of Cardiovascular Sciences, Lewis Katz School of Medicine, Temple University, Philadelphia, USA

**Keywords:** Transcriptome, Long noncoding RNA, Diabetes, Ischemic heart disease, Db/db mice

## Abstract

More than 10% of adults in the United States have type 2 diabetes mellitus (DM) with a 2–4 times higher prevalence of ischemic heart disease than the non-diabetics. Despite extensive research approaches to limit this life-threatening condition have proven unsuccessful, highlighting the need for understanding underlying molecular mechanisms. Long noncoding RNAs (lncRNAs), which regulate gene expression by acting as signals, decoys, guides, or scaffolds have been implicated in diverse cardiovascular conditions. However, their role in ischemic heart disease in DM remains poorly understood. We provide new insights into the lncRNA expression profile after ischemic heart disease in DM mice. We performed unbiased RNA sequencing of well-characterized type 2 DM model db/db mice or its control db/+ subjected to sham or MI surgery. Computational analysis of the RNA sequencing of these LV tissues identified several differentially expressed lncRNAs between (db/db sham vs. db/db MI) including Gm19522 and Gm8075. lncRNA Gm-19522 may regulate DNA replication via DNA protein kinases, while lncRNA Gm-8075 is associated with cancer gene dysregulation and PI3K/Akt pathways. Thus, the downregulation of lncRNAs Gm19522 and Gm8075 post-MI may serve as potential biomarkers or novel therapeutic targets to improve cardiac repair/recovery in diabetic ischemic heart disease.

**Supplementary Information:**

The online version contains supplementary material available at 10.1186/s12933-024-02441-6.

## Introduction

Diabetes mellitus (DM) is one of the leading chronic diseases globally, accounting for approximately 30 million cases, with an economic burden of $ 412 billion in the United States of America alone [1, 2]. Patients with diabetes mellitus are at a significantly higher risk of mortality and development of cardiovascular events compared to non-diabetic individuals. In fact, the prevalence of ischemic heart disease in diabetic patients is more than four times higher than that of non-diabetic individuals [3, 4]. Current approaches to restrict heart failure in DM patients with ischemic events are limited. Therefore, identifying novel mechanisms and therapeutic targets that can modulate ischemic heart failure in DM patients will have enormous implications for the current methods of treating patients.

Several noncoding RNAs have been implicated in diabetes and associated with heart failure [5–7]. LncRNAs are a new class of noncoding regulatory RNA, which are > 200 nt in size [8]. They regulate gene expression by acting as signals, decoys, guides, or scaffolds in modifying epigenetic, transcriptional, and post-transcriptional mechanisms [8]. Emerging evidence establish the role of lncRNAs in diabetic cardiomyopathy, such as inhibition of lncRNA zinc finger antisense 1 (ZFAS1) prevents cardiomyocyte ferroptosis and improves the progression of diabetic cardiomyopathy using db/db mice [9]. Additionally, elevated expression of lncRNA Kcnq1ot1 was observed in high-glucose treated cardiac fibroblast and diabetic myocardial tissues of streptozotocin (STZ) treated C57BL/6 mice [6]. Furthermore, suppression of Kcnq1ot1 improved cardiac fibrosis in vivo and in vitro. However a systemic analysis of lncRNA in the context of diabetic ischemic heart disease has not been well-studied.

To address this gap, we performed unbiased RNA sequencing of well-characterized DM model B6.BKS(D)-Lepr^*db*^/J (db/db) mice subjected to sham or induced myocardial infarction (MI) surgery. We investigated transcriptome-wide changes in lncRNAs with or without MI. Computational analysis of the RNA sequencing of the left ventricular (LV) tissue identified differentially expressed lncRNAs between (db/db sham vs. db/db MI) which were association with cellular and molecular pathways that regulate various diseased conditions, including diabetic ischemic heart disease.

## Materials and methods

### Animal model

The use of animals in this study followed the Guide for the Care and Use of Laboratory Animals published by the US National Institutes of Health. In addition, all experimental protocols have been approved by the Institutional Animal Care and Use Committee of Temple University School of Medicine. Eight-weeks-old leptin receptor-deficient db/+ and db/db male and female mice were purchased from Jackson Research Laboratory (Bar Harbor, ME).

### Induction of myocardial infarction

Mice were anesthetized with 2–3% isoflurane inhalation with an isoflurane delivery system. Following anesthesia, MI was induced in db/+ (5 male, 5 female) and db/db (5 male, 4 female) mice by ligation of the left anterior descending coronary artery (LAD) as described [10–12]. Mice were followed up for changes in LV cardiac function for 2- and 4-weeks post-MI, using transthoracic echocardiography as described [10–12].

### RNA isolation

Total RNA was extracted from LV tissue of db/+ and db/db mice post-sham or MI surgery using RNeasy Mini Kit (Qiagen), as per manufacturer’s protocol. All samples first underwent quality control assessment to ensure successful library preparation and sequencing. RNA sample quality was assessed by NanoDrop and Agilent 2100 BioAnalyzer. Samples with RNA integrity number (RIN) above 7, OD260/280: 2, and OD260/230 ≥ 2 were used. In addition, RNA degradation and contamination were monitored on 1% agarose gels before RNA-Seq [13].

### Library preparation and lncRNA sequencing

Library preparation for non-coding RNAs was performed based on the manufacturer’s protocol (Illumina, San Diego, USA). In brief, a total 300 ng of RNA was used to prepare the library. Quantity of prepared cDNA (complementary DNA) library was assessed with KAPA library quantification kit (KAPA Biosystem, Wilmington, USA). Further, sequencing was performed based on the manufacturer’s protocol (Illumina, San Diego, USA) by using pair-end 100 base sequencing.

### Sequencing data analysis

FASTQ files were preprocessed with fastp [14] (version 0.21.0) using the default setting. After preprocessing of sequencing reads, STAR [15] (version 020201) was used to map the reads to the reference genome (GRCm39.103). To calculate counts per million (CPM) values and derive differentially expressed genes, the R package, edgeR [16] (version 3.30.3), was used. False discovery rate (FDR)-adjusted p-values were used for further analysis.

### Data visualization and gene enrichment analyses

RNA-Seq datasets were analyzed using iDEP [17] (integrated Differential Expression and Pathway analysis), a comprehensive web-based resource that integrates 63 R/Bioconductor packages, 2 web services, alongside extensive annotation and pathway databases, accessible at http://ge-lab.org/idep/. We assessed distribution of transformed data, batch effects, and generated Principal Component Analysis (PCA) and Heatmaps to ensure that our data were appropriately normalized and visually interpretable for pathway enrichment analysis. For pathway enrichment analysis, both cis- and trans-regulatory elements were analyzed using four different databases: (1) Kyoto Encyclopedia of Genes and Genomes (KEGG) Human database (2021 version), (2) Elsevier Pathway Collection, (3) BioPlanet 2019, and (4) The Molecular Signatures Database (MsigDB) 2020. Next, X2K network was generated to map predictive interactions between proteins, kinases, and transcription factors. To further analyze the functional predictions of lncRNAs, we used lncHUB2 [18], a web server database available at https://maayanlab.cloud/lncHUB2 that takes into account co-expression correlations based on ARCHS4’s processed RNA-seq samples, expression statistics in various tissues and cell lines, predictions on biological functions, molecular pathway, and subcellular localizations.

### Real-time quantitative reverse transcription PCR

We isolated total RNA from the LV tissue of db/+ and db/db mice post-sham or MI surgery and cDNA synthesis was performed using the cDNA synthesis kit (Cat # 4368813, Applied BioSystems, Waltham, MA, United States). Real-time polymerase chain reaction (RT-qPCR) was performed using the SYBR (Power Up SYBR Green Master Mix, Cat #A25742, Applied BioSystems, Waltham, MA, United States) and QuantStudio™ three Real-Time PCR. Primer sequences used are presented in supplementary Table 1.

### Statistical analysis

Results are presented as mean ± standard error of the mean (SEM). Data were analyzed using one way ANOVA followed by Tukey test for comparison of groups. Student t-test (two-tailed) was performed while comparing two groups. Statistical analysis was performed using GraphPad Prism 9, version 9.2.0 (GraphPad Software, Inc., La Jolla, CA, United States). Differences were considered statistically significant at *p* < 0.05.

## Results

### Establishment of MI in db/db mice

**Fig. 1:**
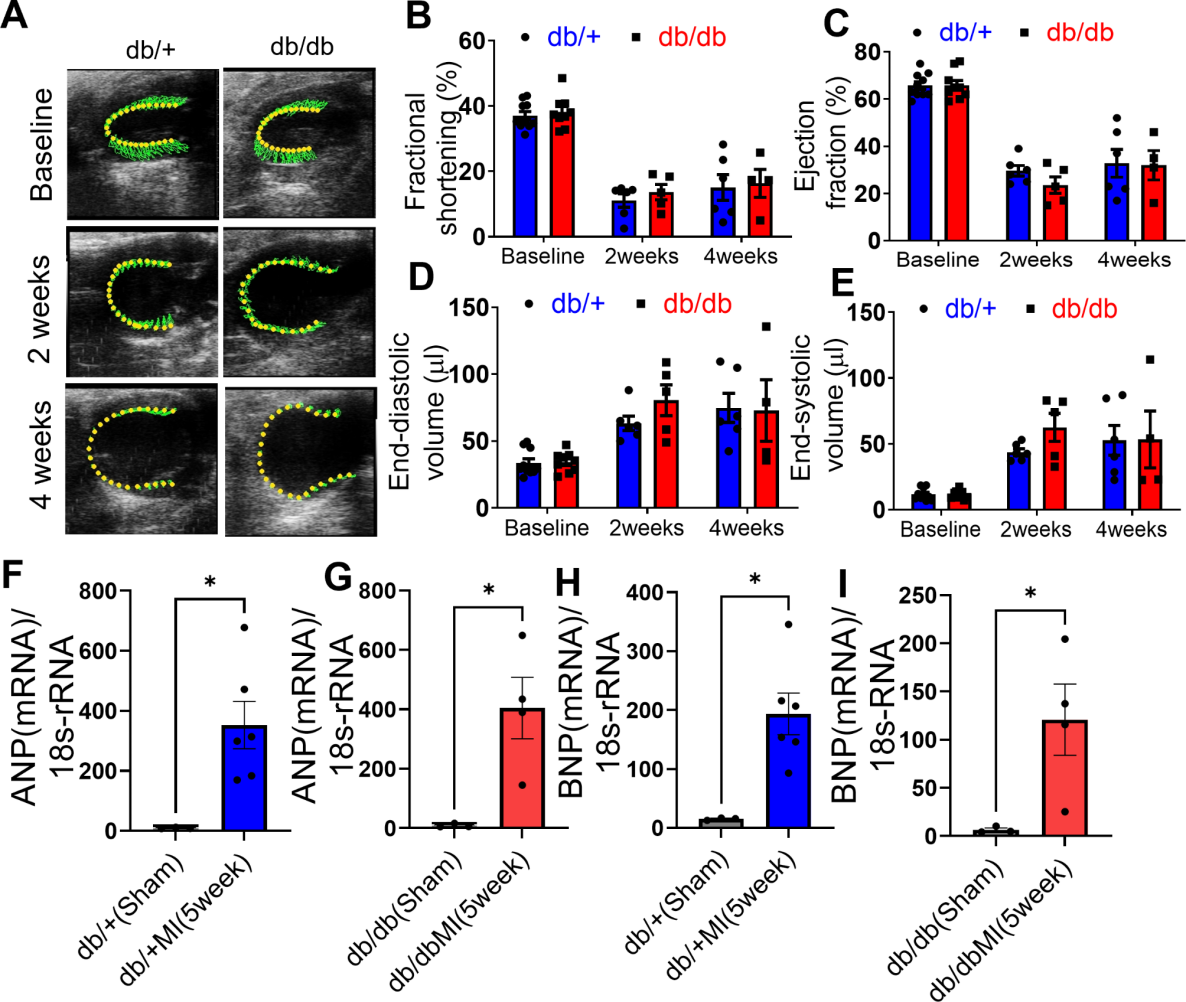
Establishment of heart failure model. M-mode representative images (**A**) depicting cardiac performance followed by the Fractional shortening (%) (**B**), ejection fraction (%) (**C**), end-diastolic volume (µL) (**D**), end-systolic volume (µL) (**E**) measurements in db/+ db/db mice before and after MI. mRNA expression of Anp (**F**–**G**) and Bnp (**H**–**I**) gene in db/+ and db/db mice hearts in sham and 5 weeks post-MI. Data is represented as Mean ± SEM, with 18s rRNA used as a housekeeping control for normalization. Two-tailed unpaired student t-test was used to compare the statistical significance among groups. *p < 0.05

We confirmed MI induction by echocardiography analysis in both cohorts of mice, including db/+ and db/db at 2 weeks and 4 weeks of post MI surgery. We observed MI-associated mortality rate of 40% in db + group and, as expected, a higher mortality rate of 56% in db/db mice at four weeks post-MI. Cardiac dysfunction was observed in these mice with reduced ejection fraction as well as increased end diastolic and systolic volume (Fig. 1A–E). The mRNA expression of atrial natriuretic peptide (*Anp*) and brain natriuretic peptides (*Bnp*), hallmarks of a failing heart [19], were evaluated by qRT-PCR at 5 weeks post-MI surgery, which revealed significant increases in these markers in both animal groups (Fig. 1F–I). This data shows we successfully induced MI in both groups of mice, and increased heart failure markers. Db/db MI mice serves as a model of diabetic ischemic heart disease study in this paper (Supplementary Table 2).

### Identification of differentially regulated lncRNA in LV tissue of db + or db/db hearts with or without MI

**Fig. 2:**
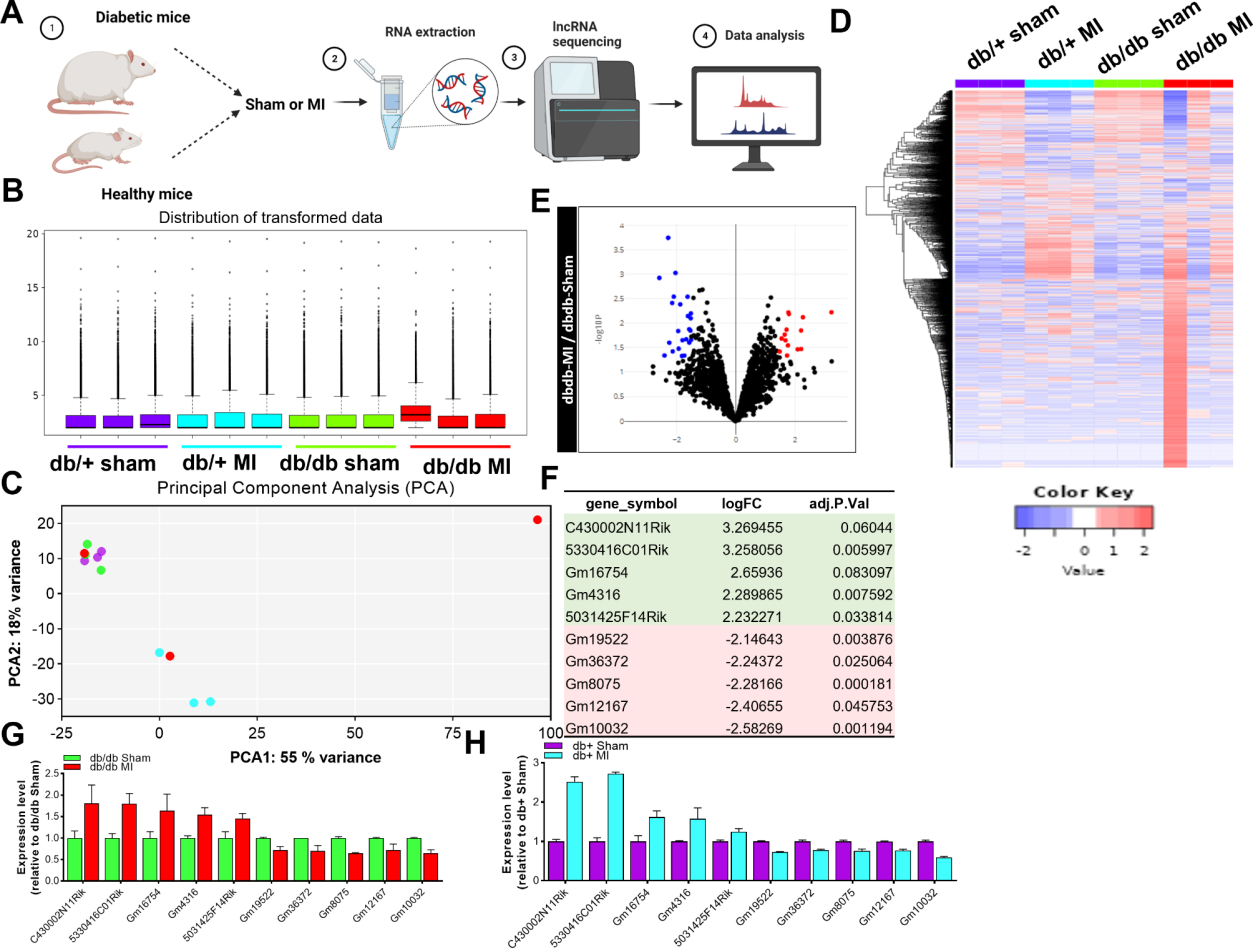
LncRNA signature in LV tissue of db + or db/db hearts with or without MI. **A** Schematic outline of experimental design and downstream bioinformatic analysis. **B** Distribution of transformed RNA sequencing data. **C** Principal component analysis of RNA sequencing data. **D** A heat map of lncRNAs differentially expressed at 3 days following left anterior descending coronary artery (LAD) artery ligation or sham surgery in db + or db/db mice showing distinct profiles. **E** Volcano plots showing Log_2_-fold changes for the differentially regulated lncRNA in the LV tissue of db + or db/db hearts at 3 days post sham or MI surgery. Red dots indicate significantly up-regulated lncRNAs; blue dots indicate down-regulated lncRNAs. **F** A table showing top five up or down-regulated lncRNAs in the LV tissue of db + or db/db hearts at 3 days post sham or MI surgery. **G**–**H** Normalized counts of the top 5 up or downregulated lncRNA in the LV tissue of db + or db/db hearts at 3 days post sham or MI surgery. Data are shown as Log_2_ fold change > 2, *p* < 0.001, and FDR < 0.05

RNA isolated from the LV tissue of diabetic mice db/db and db/+ that underwent myocardial infarction surgery followed by deep RNA sequencing for lncRNA (Fig. 2A–D). Upon analysis, we identified several lncRNA exhibited differential expression. Bioinformatic analysis of sequencing data of db/db mice post MI revealed differentially expressed lncRNAs in LV tissue of mice (Fig. 2E). We chose to analyze the top 5 upregulated and 5 downregulated lncRNA in the heart of db/db mice post MI (Fig. 2F–H).

### Validation of lncRNAs GM19522 and GM8075 in diabetic mouse hearts post-MI

**Fig. 3:**
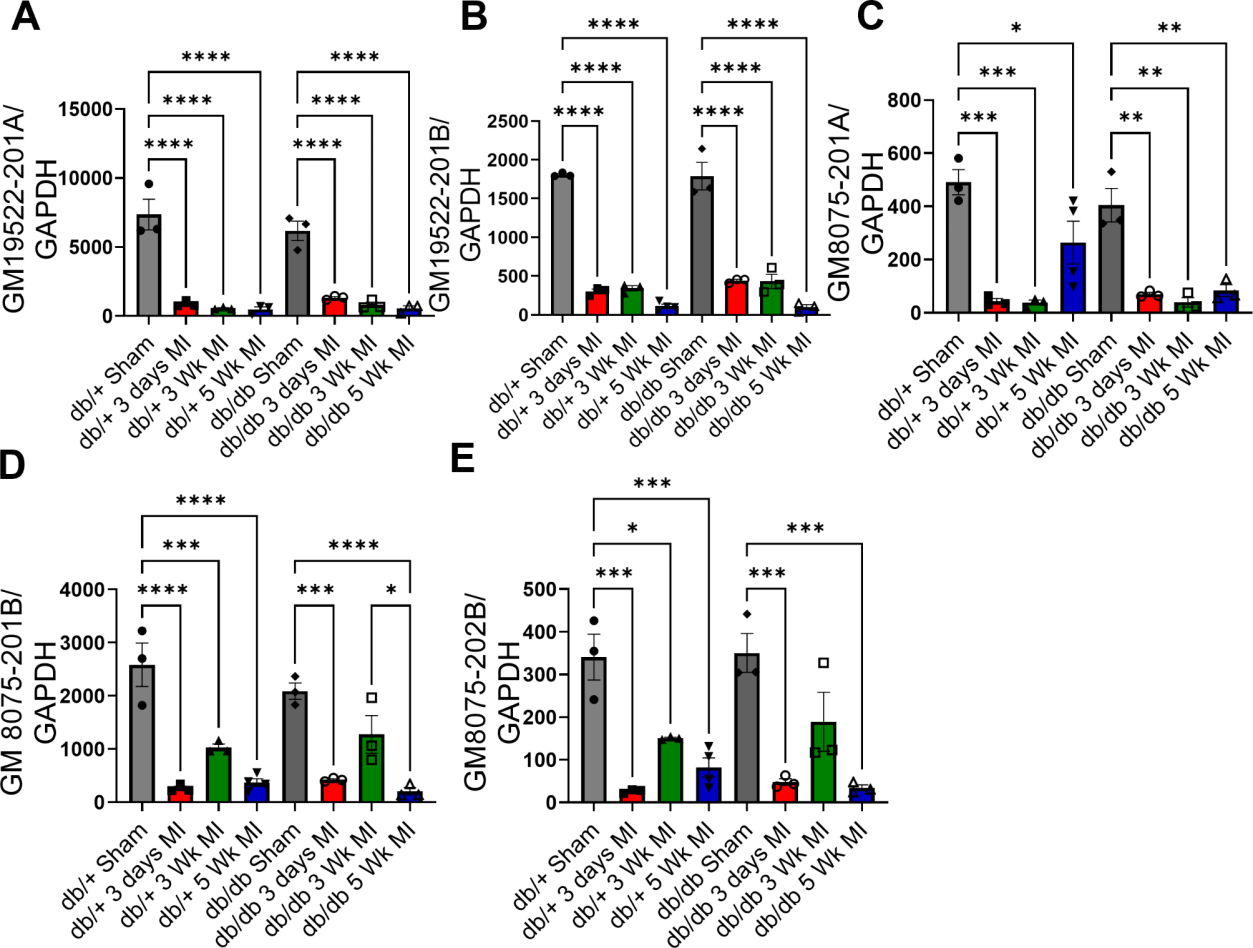
Validation of LncRNAs GM19522 and GM8075 in MI hearts of diabetic mice. Quantitative real-time PCR data showing downregulation in the expression of lncRNA GM19522-201 A (**A**), GM19522-201B (**B**), GM8075-201 A (**C**), GM8075-201B (**D**), and GM8075-202B (**E**) in sham or MI mice(db/+ or db/db) at 3 weeks and 5 weeks compared to sham surgery. *Gapdh* was used as housekeeping control. Data is represented as Mean ± SEM, calculated using. One-way ANOVA was used to compare the statistical significance among groups. *p < 0.05, **p < 0.01, ***p < 0.001, and ****p < 0.0001

To the top two downregulated markers from the bioinformatic analysis Gm19522 and Gm8075, were selected for expression validation using real-time PCR. Gm19522-201 A and Gm19522-201B were significantly downregulated in post MI on day 3, week 3 and week 5 compared to sham mice (Fig. 3A, B). Similarly, the expression of Gm8075-201 A, Gm8075-201B and Gm8075-202B was significantly downregulated in post MI mice on day 3, week 3 and week 5 when compared to sham control mice (Fig. 3C–E). These results conform with bioinformatic analysis (Fig. 2E, H).

### Pathway enrichment analysis

**Fig. 4:**
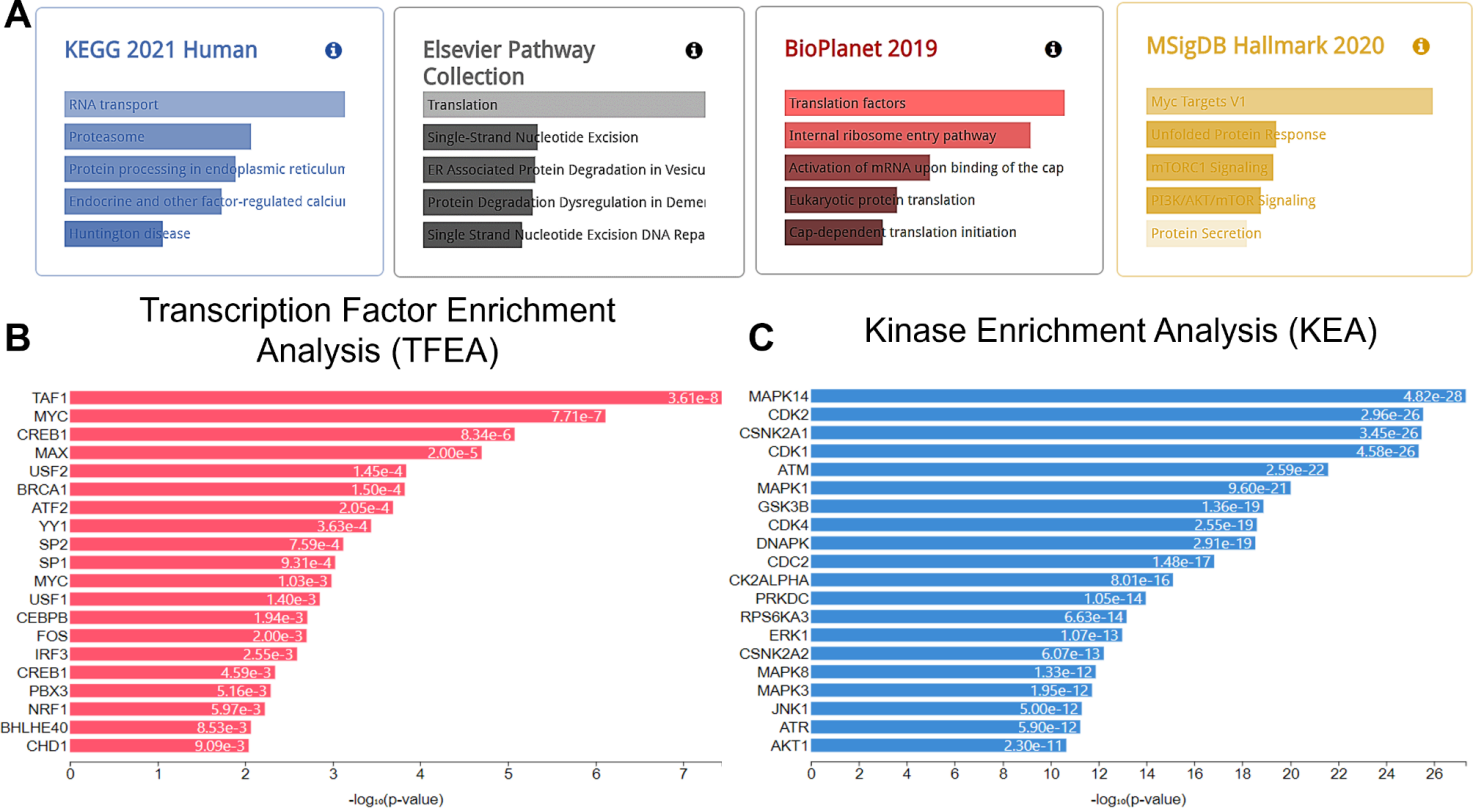
Pathway enrichment analysis. **A** A comprehensive analysis to identify downstream target genes of (*cis*- and *trans*-regulatory) of the differentially expressed lncRNAs using multiple databases 2021 Kyoto Encyclopedia of Genes and Genomes (KEGG) Human database, Elsevier Pathway Collection, BioPlanet 2019, and the Molecular Signatures Database 2020 (MsigDB). **B** lncRNAs potential downstream transcription factors and kinases analysis performed using Transcription factor enrichment analysis (left panel), and **C** identified key kinases involved via kinase enrichment analysis (right panel)

To better understand the molecular pathways and biological functions associated with these lncRNAs, we performed pathway enrichment analysis using several databases, including the KEGG 2021 human database, Elsevier pathway collection, BioPlanet 2019, and MSigDB 2020 (Fig. 4A) [13]. Our results suggest that pathways related to RNA transport, protein synthesis, protein transport, and degradation may be dysregulated (Fig. 4A). Furthermore, we evaluated the downstream association of lncRNAs to transcription factors and associated kinases by performing Transcription Factor Enrichment Analysis (TFEA) (Fig. 4B) and Kinase Enrichment Analysis (KEA) (Fig. 4C). TEFA analysis uncovered important transcription factors, including TAF1, MYC, CREB1, MAX, and USF2 (Top 5 listed here) (Fig. 4B), while KEA analysis revealed changes in MAPK14, CDK2, CSNK2A1, CDK1 and ATM (Top 5 listed here) (Fig. 4C).

### The eXpression2Kinases (X2K) network displays predictive interactions of proteins, kinases and transcription factors

**Fig. 5:**
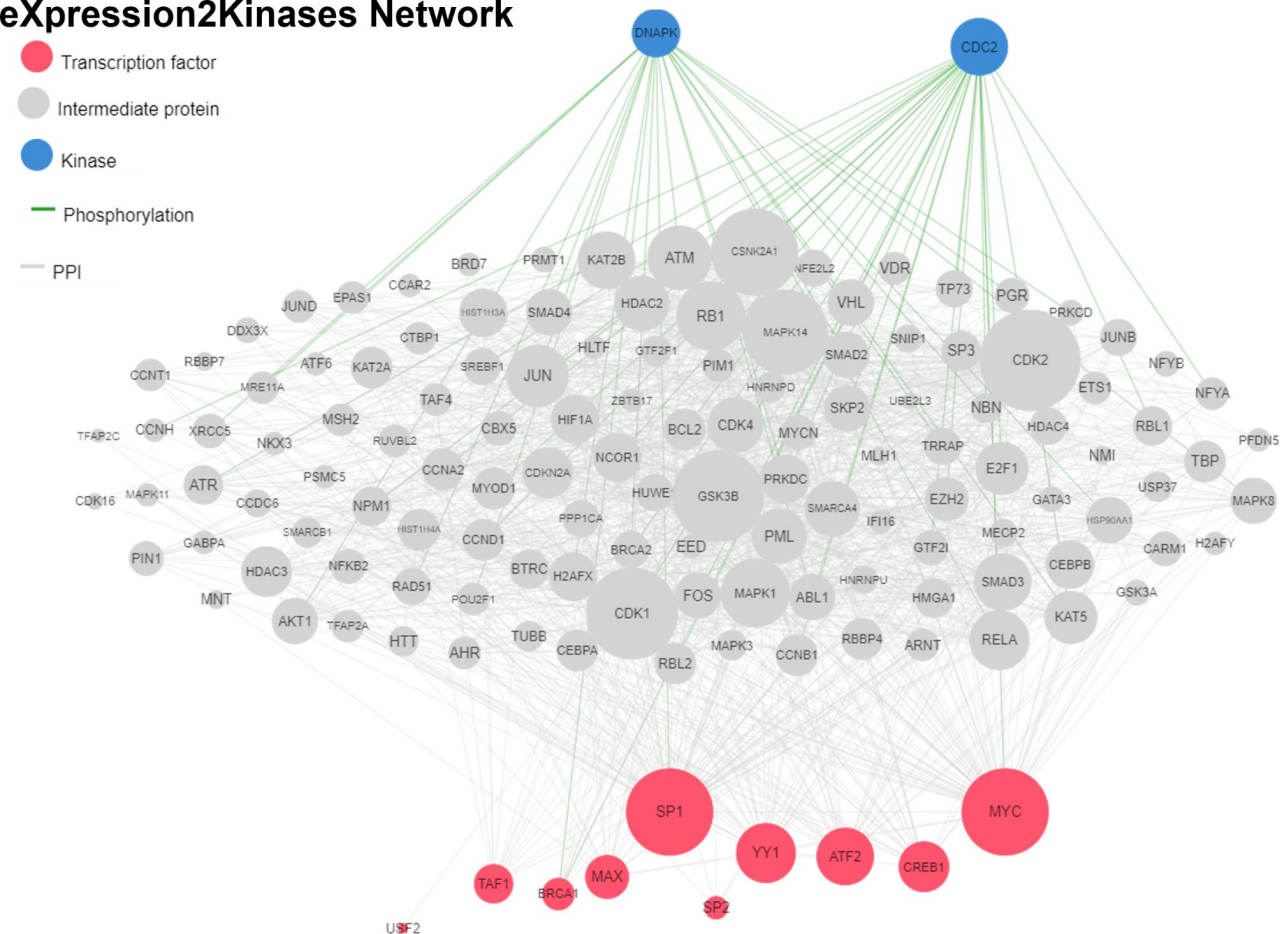
The eXpression2Kinases (X2K) network analysis and predictive interactions. The X2K network illustrates the predictive interactions of proteins, kinases, and transcription factors. Pink nodes represent the top transcription factors predicted to regulate the expression of the input gene list; blue nodes represent the top predicted protein kinases known to phosphorylate the proteins within the expanded subnetwork. Green network edges/links represent kinase-substrate phosphorylation interactions between kinases and their substrates, while grey network edges represent physical protein-protein interactions

Analysis of TFEA and KEA facilitated the construction of a network illustrating the interactions between transcription factors and kinases influenced by the dysregulated lncRNAs.The analysis of eXpression2Kinases (X2K) network revealed that SP1, MYC, MAX, ATF2, and CREB1 are the most affected transcription factors (Fig. 5, denoted in pink color) are associated with CDC2 and DNAPK kinases (Fig. 5, denoted in blue color).

## Discussion

Current investigation aims to establish the link between the expression of lncRNAs and their potential role in diabetic ischemic heart diseases. In recent years, lncRNAs have emerged as potential biomarkers in diabetic heart diseases [7, 20–22]. One of the recent findings by Pant et al., reported that five Lnc RNAs including, XLOC015617, AK035192, Gm10435, TCR-α chain, and MouselincRNA0135 were found deregulated in mouse model of diabetic cardiomyopathy [22]. In this study, we provide first evidence showing significant downregulation in the expression of lncRNAs (Gm 8075 and Gm 19522) with the progression of MI in diabetic mice. Although there is currently no literature specifically addressing these lncRNAs in the context of diabetic ischemic heart disease. Our results suggest they may have an involvement in key molecular pathways in the disease process. Future studies are warranted to determine functional signifance of these lncRNAs in diabetic ischemic heart disease. Our study provides a novel basis for further investigation into their potential role as biomarkers, therapeutic targets or in novel mechanisms. By exploring novel lncRNAs we may identify new insights into pathophysiology of MI in diabetes which would ultimately aid developing innovative approaches to diagnose or treat diabetic heart disease.

Interestingly, our computational data analysis showed that these lncRNAs are involved in the regulation of various molecular pathways, including RNA transport, protein synthesis, and degradation. Our TEFA and KEA analysis establishes a link between transcription factors and kinases associated with lncRNAs. LncRNAs are known to modulate biological functions at cellular and molecular levels by regulating various genes [8, 20]. LncRNAs can also affect the expression pattern of kinases and affect the outcome of the biological processes [23]. In the current study, we investigated the interaction between transcription factors and kinases post-analysis of TFEA and KEA and found that CDC2 and DNAPK were the most affected kinases that showed the relationship with various transcription factors, including SP1, MYC, ATF2, YY1, and CREB1 (Fig. 5; transcription factors are highlighted in pink whereas kinases are highlighted in blue). These factors regulate the cell cycle, DNA damage response, and transcriptional control. For example, CDC2 is crucial for cell cycle progression and proliferation in vascular smooth muscle cells and cardiomyocytes. Overexpression of CDC2 promotes cardiomyocyte proliferation [24, 25], an important process in cardiac repair mechanisms post-injury. Additionally, CDC2 is implicated in the hyperplastic proliferation of vascular smooth muscle cells in pulmonary arterial hypertension [26], which could lead to vascular obstruction and elevate blood pressure in the pulmonary circulation, and lead to right heart failure. In diabetic ischemic heart disease, abnormal activation of CDC2 may cause maladaptive vascular and cardiac remodeling by promoting excessive proliferation or disrupting normal cell cycle control in cardiomyocytes. DNA-PK helps maintain genomic stability by repairing DNA double-strand breaks during cell division, thus preventing mutations and chromosomal abnormalities. In cardiomyocytes, activation of DNA-PK during ischemic conditions exacerbates mitochondrial dysfunction and apoptosis, contributing to pathological remodeling and cardiac dysfunction [27]. Other studies showed that DNA-PK contributes to vascular proliferative disease processes such as neointimal formation in atherosclerosis-prone mice [28]. Similarly, the transcription factor SP1, involved in cell growth, differentiation, and DNA damage response, has been associated with hypertrophic cardiomyopathy by regulating the expression of genes critical for structural integrity and mitochondrial function, essential for cardiomyocyte function under stress [29]. Finally, MYC has been extensively described for its role in cellular metabolism and growth by enhancing glucose metabolism and mitochondrial biogenesis in cardiomyocytes, which could initially be a beneficial adaptive mechanism in response to acute stress [30–32]. However, persistent myc activation in the context of chronic stress could lead to hypertrophic cardiomyopathy and heart failure. In cardiac cells, CREB1 regulates genes essential for cell survival, metabolism, and mitochondrial function. Cardiac-specific overexpression of dominant-negative CREB leads to increased mortality and mitochondrial dysfunction in female mice [33]. Its dysregulation in diabetes can lead to mitochondrial dysfunction and cell death, processes central to various cardiovascular diseases, including myocardial infarction and heart failure. The metabolic disturbances and hyperglycemia in diabetic ischemic heart disease might alter the activity of kinases and transcription factors like DNA-PK, CDC2, MYC, SP1, and CREB1, exacerbating cardiac dysfunction. These interactions highlight the complexity of diabetic ischemic heart disease pathogenesis and provide new opportunities to explore how these therapeutic targets are regulated to improve cardiac function in diabetes.

Importantly, our KEGG analysis suggested the role of lncRNA Gm-19522 in various molecular processes, including DNA replication and repair, indicating a possible involvement of DNAPK (Supplementary Fig. 1). Interestingly, DNAPKs are known to the regulate cardiomyocyte-specific mitochondrial apoptosis in post ischemia [27]. However, lncRNA Gm-8075 showed involvement in the dysregulation of genes of various cancers, including gastric cancer, breast cancer, and melanoma, as well as other metabolic pathways like PI3K/Akt. However, future studies are warranted to tease out the missing links of molecular mechanism that govern the relationship between lncRNAs, kinases and transcription factors. Future studies are warranted to determine lncRNAs interactome in diabetic ischemic heart disease.

Db/db mice are a widely used model for studying diabetes induced cardiovascular disease [34]. They develop diastolic dysfunction without additional stressors [35]. In our study, 10-12-week-old db/db sham injury mice exhibited significant changes in lncRNA expression, including Gm43332 (upregulated) and Gm5420 (downregulated) compared to db + sham mice (Supplementary Fig. 2). Further KEGG pathway analysis revealed their potential roles in fatty acid biosynthesis, oxidative phosphorylation, protein digestion/absorption, mucin type O-glycan biosynthesis, and N-glycan biosynthesis. Further research is warranted to understand the specific roles of these lncRNAs in diabetes induced diastolic dysfunction.

## Conclusions

In summary, our current study highlights downregulation of lncRNAs Gm19522 and Gm 8075 expression post-MI in diabetic mice. These findings suggest that Gm19522 and Gm8075 may serve as potential biomarkers or as potential therapeutic targets for improving cardiac repair/recovery in diabetic ischemic heart disease.

## Electronic supplementary material

Below is the link to the electronic supplementary material.

Supplementary Figure-1: KEGG and ChEA analysis of our top target lncRNAs Gm19522 and Gm8075 in db/db MI vs db/db sham mice.

Supplementary Figure-2: Volcano plots showing Log2-fold changes for the differentially regulated lncRNAs in the LV tissue of db/+or db/db hearts without MI.

Supplementary Table 1: Primers sequences used in this study.

Supplementary Table 2: Echocardiographic measurements.

## Data Availability

“Data is provided within the manuscript or supplementary information files”.

## Abbreviations

Anp: Atrial natriuretic peptide
Bnp: Brain natriuretic peptide
cDNA: Complementary DNA
DM: Diabetes mellitus
FDR: False discovery rate
IDEP: Integrated Differential Expression and Pathway Analysis
KEA: Kinase Enrichment Analysis
KEGG: Kyoto Encyclopedia of Genes and Genomes
LAD: Left anterior descending
lncRNA: Long non-coding RNA
LV: Left ventricle
MI: Myocardial infarctiom
MsigDB: Molecular Signatures Database
PCA: Principal Component Analysis
RIN: RNA integrity number
RT-qPCR: Real-time polymerase chain reaction
SEM: Standard error of the mean
TFEA: Transcription Factor Enrichment Analysis
X2K: eXpression2Kinases
ZFAS1: Zinc finger antisense 1
