# Supplementary figures and images for: Transcriptome wide changes in long noncoding RNAs in diabetic ischemic heart disease
Source: Cardiovasc Diabetol. 2024 Oct 17;23:365. doi: 10.1186/s12933-024-02441-6 (PMC11488282; doi:10.1186/s12933-024-02441-6)

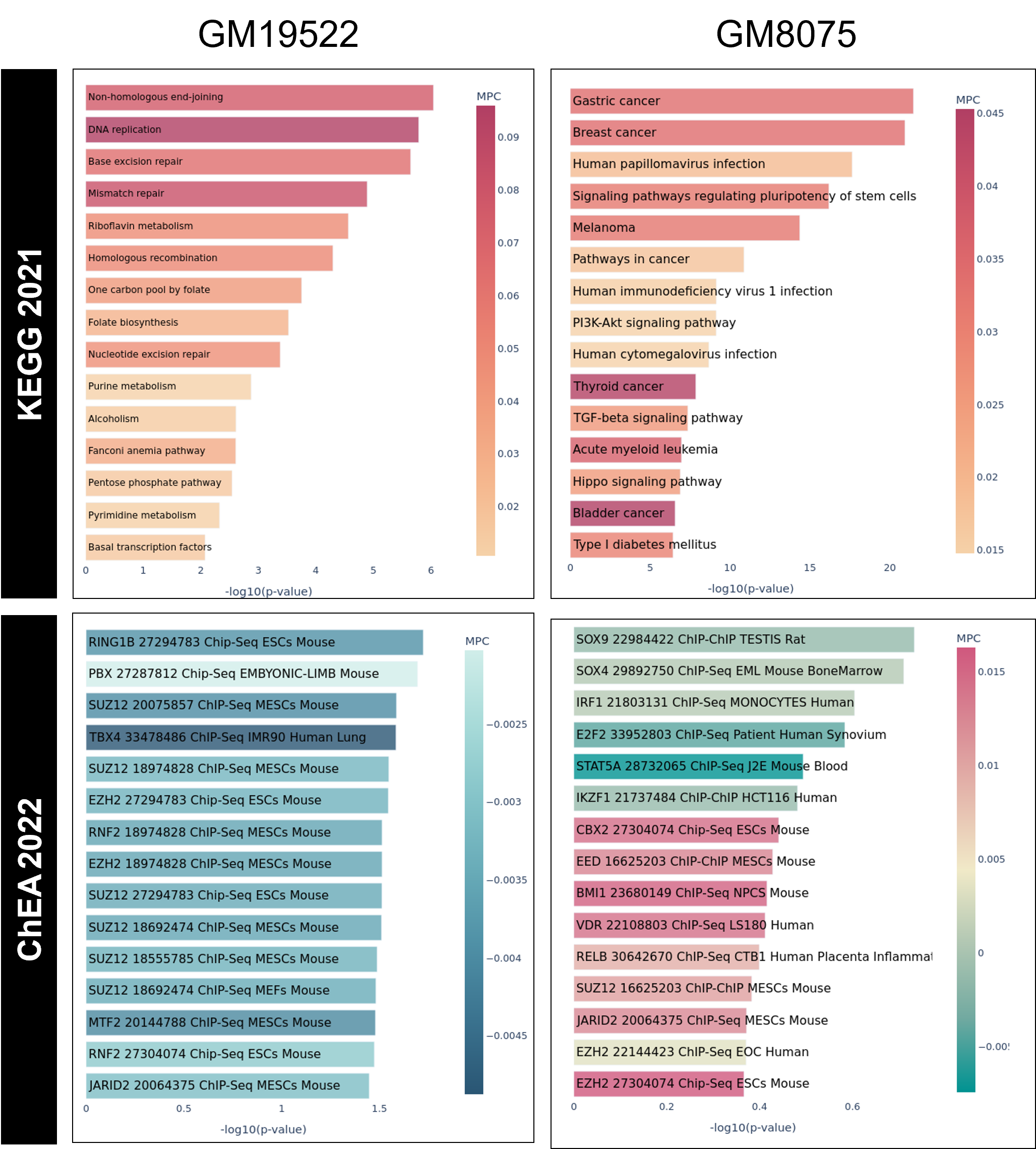

Supplement: Supplementary file 1 — Supplementary Figure-1: KEGG and ChEA analysis of our top target lncRNAs Gm19522 and Gm8075 in db/db MI vs db/db sham mice. [file 12933_2024_2441_MOESM1_ESM.tif]

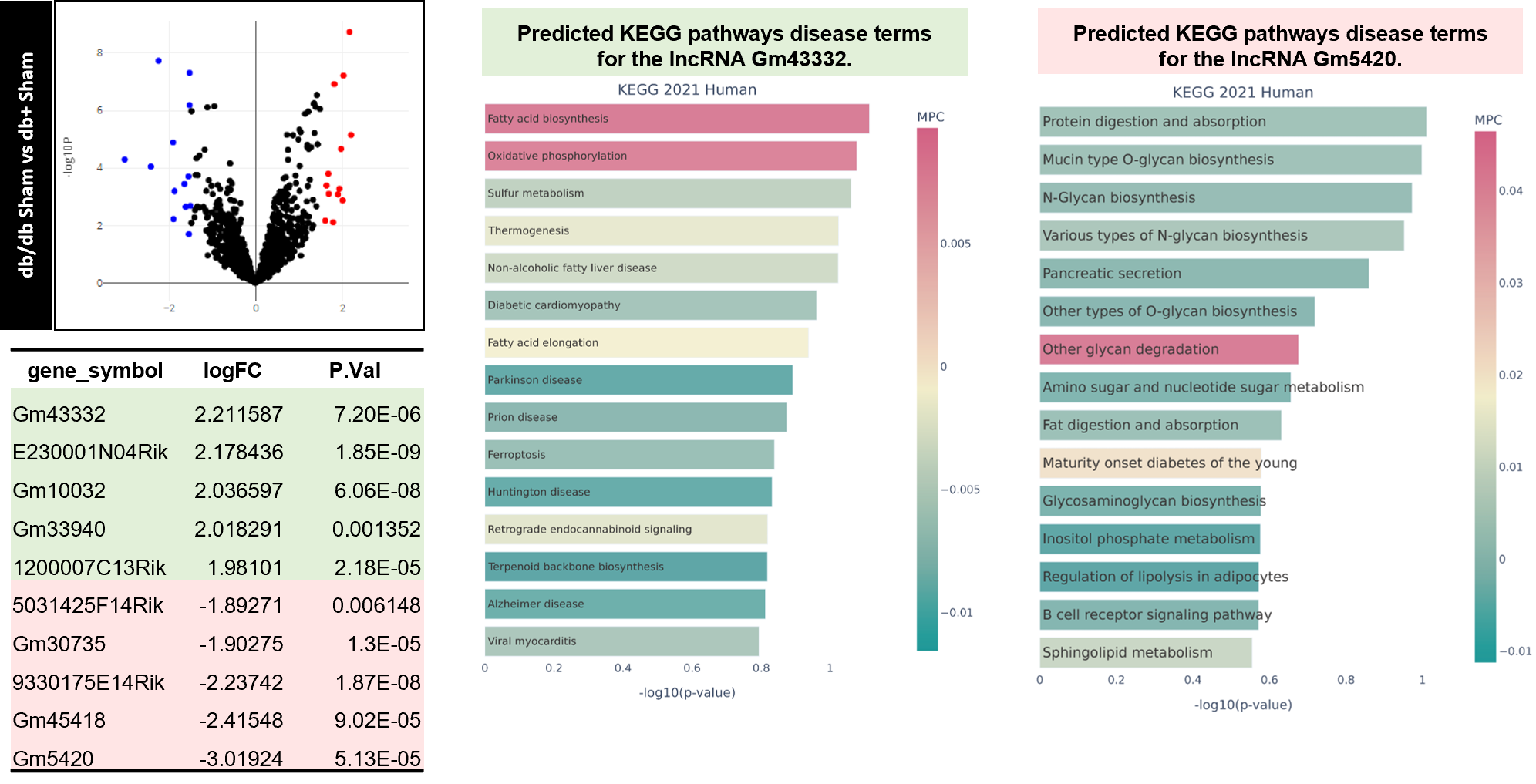

Supplement: Supplementary file 2 — Supplementary Figure-2: Volcano plots showing Log2-fold changes for the differentially regulated lncRNAs in the LV tissue of db/+or db/db hearts without MI. [file 12933_2024_2441_MOESM2_ESM.tif]
